# Assessment of Natural Radioactivity in Cements Used as Building Materials in Poland

**DOI:** 10.3390/ijerph191811695

**Published:** 2022-09-16

**Authors:** Sylwia Lewicka, Barbara Piotrowska, Aneta Łukaszek-Chmielewska, Tomasz Drzymała

**Affiliations:** 1 Faculty of Safety Engineering and Civil Protection, The Main School of Fire Service, 52/54 Słowackiego Street, 01-629 Warsaw, Poland; 2 Central Laboratory for Radiological Protection, 7 Konwaliowa Street, 03-194 Warsaw, Poland

**Keywords:** cement, natural radioactivity, radiation hazard parameters, statistical analysis

## Abstract

It has been analyzed in this article the radioactivity concentrations of ^226^Ra, ^232^Th, ^40^K and radiological hazard parameters in different types of cements commonly used in Poland and available on the Polish market. The radiological hazard parameters are, in particular, absorbed gamma dose rate, annual effective dose, radium equivalent activity, the external hazard index, and the gamma and alpha indices. The radionuclide activities of the most important radionuclides ^226^Ra, ^232^Th, ^40^K have been determined by gamma-ray spectrometry with the use of two kinds of spectrometers of different operational parameters. One performed also measurements on 30-day and 45-day aged samples as to verify if there is a statistically significant difference in radioactivity concentration for shorter and longer aging time. The radioactivity concentrations in the cement samples ranged from 21.7–75.7 Bq·kg^−1^ for ^226^Ra, 12.3–47.3 Bq·kg^−1^ for ^232^Th to 123–430 Bq·kg^−1^ for ^40^K. The radiological parameters in cement samples were calculated as follows: mean radium equivalent activity *Ra_eq_* = 127 Bq·kg^−1^, mean absorbed gamma dose rate *D* = 115 nGy·h^−1^, mean annual effective dose *E* = 570 µSv·y^−1^, external hazard index *H_ex_* = 0.32, internal hazard index *H_in_* = 0.51, mean activity concentration index *I_γ_* = 0.47 and mean alpha index *I_α_* = 0.28. The results were compared with the reported data from other countries and the international standard values given by European Commission (EC) and United Nations Scientific Committee on the Effects of Atomic Radiation (UNSCEAR 2000). Finally, thorough statistical analysis has been performed.

## 1. Introduction

Natural radiation background is an inherent element of life on Earth. Its sources comprise cosmic radiation and Earth radiation. The biggest contributions to the natural Earth radiation background in the human environment are radioactive decays occurring in soils (one can estimate that gamma radiation reaches the surface from soils mainly as it has the greatest penetration), as well as radon produced in the lithosphere through the alpha decay of Ra-226, and penetrating through the fissures to the earth’s surface. Therefore, these contributions are made by such elements, as potassium (^40^K), thorium (^232^Th) and uranium (^238^U) and its decay products, such as radium (^226^Ra) and radon (^222^Rn) [1,2]. Inside buildings, an additional source of the natural background ionizing radiation comprises natural radioactive elements—radium, thorium and potassium, present in construction elements obtained from minerals, as well as ash and slag additives to cements.

Many materials from all over the world contain various amounts of naturally occurring radioactive isotopes, and these include also building materials, which ingredients are rocks, minerals, sand, ash etc. In addition, the earth’s crust, air, water and food contain the radioactive isotopes; this is why they all are called naturally occurring radioactive materials (NORM). The main radionuclides in NORMs are the long-lived isotopes from the three natural radioactive series, and these are radium Ra-226, Ra-224, thorium Th-232, uranium U-235, U-238. This also includes potassium K-40 whose origin is cosmic [1]. Therefore, various international organizations such as: ICRP 1994 [3]; European Commission 1999 [4]; UNSCEAR (2000, 2008) [1,5]; and The Council of the European Union 2014 [6] introduced the standards of radiation protection in order to protect the population from the negative effects of ionizing radiation. Typical concentrations of natural radioactive isotopes ^40^K, ^226^Ra and ^232^Th in building materials are 500, 50 and 50 Bq kg^−1^, respectively [5]. More and more, in order to dispose industrial wastes (of which more than half is waste generated in the mining industry, i.e., slags and ashes), they are being incorporated into building materials. The presence of these additives can significantly increase the radioactivity of materials and contribute significantly to the exposure of people to external and internal ionizing radiation emitted by them, as on average people spend about 80% of their time indoors [2]. External exposure originates from ^40^K, ^226^Ra, ^232^Th radionuclides and their decay products emitting gamma radiation, while internal exposure is caused by inhalation of radon (^222^Rn, see e.g., [7]), thoron (^220^Rn, for review see [8]) and their decay products, which, in turn, are alpha emitters.

Cement is the basic material used in all types of construction [9]. It is used in the form of mortars for joining elements, as a basic component of concrete mix, for the production of prefabricated concrete elements, large-size monolithic structures, roof tiles, bricks, etc. Such produced concrete composites are characterized by easy shaping, assembly and disassembly of elements, and are durable. Concrete is resistant to environmental conditions, it is characterized by low water absorption and high water-resistance. It can be resistant to corrosion and fire [10,11,12,13,14]. Cement itself is a binder produced by firing limestones, marls and clays in rotary kilns at a temperature of 1450 °C, from which cement clinker is formed in the first stage of production. Then, the resulting sinter is ground with lime sulphate (gypsum), which acts as a setting time regulator. During sinter grinding, the so-called non-clinker cement components act as fillers, which are often waste substances from various industries. Because the production of clinker is highly energy-consuming and is associated with high CO_2_ emissions to the air, the use of waste additives that not only do not deteriorate but in some cases improve its technical parameters becomes very popular.

The main waste additives used in the production of cements are blast furnace slag and fly ash [15]. Granular blast furnace slag is an industrial by-product that results from the production of pig iron in a blast furnace. The basic raw materials inserted to the blast furnace are: iron ores, e.g., magnetite, hematite, limonite or siderite; coke as an energy carrier; calcined bauxite; and crushed limestone and silica as fluxes. The composed set of input raw materials is subjected to thermal treatment at a temperature ranging from 1400 to 1650 °C. During the smelting of the load, liquid blast furnace slag is formed in the upper part of the pig iron, which then undergoes the process of cooling and granulating.

Fly ash is produced by burning crushed coal in furnaces of power plants or thermoelectric power stations, and then leaves the dust furnace together with the flue gases. It is in the form of fine mineral dust and consists of: basic components (SiO_2_, Al_2_O_3_, Fe_2_O_3_, CaO); secondary components (MgO, SO_3_, Na_2_O, K_2_O); trace components (TiO_2_, P_2_O_5_, Mn and others); and unburned carbon (glow losses). The essential methods of dividing fly ash are based on: furnace structure (ash from combustion in conventional boilers and ash from combustion in fluidized bed boilers); the geochemical origin of the combusted coal fuel (ashes from the combustion of hard coal and from the combustion of lignite); the selective method of collecting ashes from various sections of electrostatic precipitators (ashes from zone I, zone II and zone III); and the share of the main components (silicate, aluminum, calcium).

Among the waste substances that are currently used in modern cement plants, one can also mention alternative ecological fuels, which are used in the processes of co-combustion of traditional fuels in furnaces. Incineration of waste in this place causes heat recovery, and ashes and slags resulting from their incineration in a cement kiln become part of the raw material mixture of the clinker burnt. The alternative fuel is mainly obtained from plastics that are not recyclable. These are often also residues and waste from production processes of various industrial sectors, such as automotive, paper, food, packaging and furniture production. Alternative fuels are worn-out furniture and other bulky waste as well as all kinds of biomass: waste from wood processing, sewage sludge and residues from agricultural production.

The specific composition of the final cement product determines the way of the division of cements. The cement containing the least amount of ingredients is Portland cement (denoted as CEM-I) and consists only of cement clinker and gypsum in an amount of up to 5%. CEM-II, multi-component Portland cement, contains up to 35% of mineral additives in its volume, which may be, successively: blast furnace slag (S), silica fly ash (V), calcium fly ash (W), silica dust (D), Natural pozzolana (P), fired pozzolana (Q) and limestone (L, LL). Another type of cement is CEM-III, which is metallurgical cement. It is obtained from: Portland clinker, setting time regulator and granular blast furnace slag, the content of which ranges from 35 to 95%. This cement is more sulfate-resistant than Portland cement. Similarly to the composition of metallurgical cement, another type of common cement, with the designation CEM-IV, is pozzolanic cement, and in its composition, in addition to the basic components, contains significant additives of pozzolana. CEM-V, a multi-component Portland cement, apart from its basic components, in its volume contains significant amounts of blast furnace slag and pozzolans. Taking into account the multitude of both cements and additives used thereto, as well as the possibility of penetration into the final product of unidentified radioactive radionuclides of natural or waste origin, it is necessary to monitor the concentration of natural radioactive radionuclides of potassium (^40^K), radium (^226^Ra) and thorium (^232^Th) in cements available on the market.

**Figure 1:**
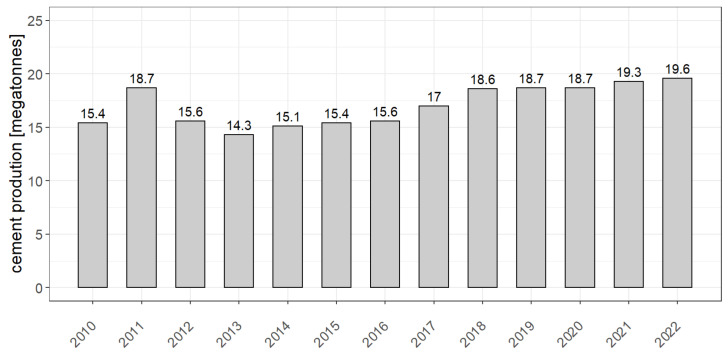
Sales of cement in Poland in the years 2010–2022. Source: https://www.polskicement.pl/aktualnosci/produkcja-cementu-w-polsce-rosnie-niestety-import-z-bialorusi-tez/ (accessed on 24 July 2022).

It should be emphasized that the global usage of cement keeps growing in a dynamic way. In 2017, the total production was estimated at 4.1 billion tones, 56.5% of which in global production belongs to China. At present, Poland is the third producer of cement in Europe, occupying the position after Germany and Italy. Over the years, cement production in Poland has undergone various fluctuations, nonetheless, since 2013 it has been steadily increasing, reaching 19.6 Mt in 2022 (according to estimates of the Statistics Poland, see Figure 1).

The present production of cement in Poland relates to a consumption of ca. 500 kg per 1 inhabitant (the average value in Europe is 309 kg, and in China 1705 kg). Such a result was achievable thanks to the advantageous macroeconomic situation and dynamic growth of construction and assembly production. Therefore, cement plays a key, yet often unnoticeable, role in human life. It is mainly used as a binder in concrete, which in turn is the starting material in construction. The knowledge of the concentrations of natural radioactive radionuclides, namely radium, thorium, and potassium in building materials is very important due to the fact that people spend 80% of their time indoors in residential buildings [2].

Assuring safe sanitary and health conditions inside premises of residential buildings requires eliminating the use of building products that contain excessive concentrations of natural radioactive radionuclides. For this reason, in various parts of the world concentrations of natural radioactive radionuclides in construction materials are under constant monitoring, i.e., in Australia [16], Austria [17], Tanzania [18], Bangladesh [19], Brazil [20], and China [21]. In Poland, the concentration of natural radioactive radionuclides radium (^226^Ra), thorium (^228^Th) and potassium (^40^K) in construction materials is being monitored, as well [22,23]. They serve as a basis for assessing the suitability of materials in the construction sector. Those elements are primarily the main emitters of alpha, beta, and gamma radiation and are not neutral to human health.

The article presents concentrations of natural radioactive radionuclides of radium (^226^Ra), thorium (^232^Th), and potassium (^40^K) for 15 various types of cements, commonly used in the construction industry, which are available on the Polish market. In order to determine exposure to ionizing radiation, a calculation was made of the gamma radiation dose rate (*D*), annual effective dose (*E*), radium equivalent activity (*R_eq_*), external hazard index (*H_ex_*) and internal hazard index (*H_in_*), the index of exposure to gamma and alpha radiation (*I_γ_*) and (*I_α_*), respectively. The obtained results were then compared with international standard values defined by the European Commission [4], and values recommended by the United Nations Committee on the Effects of Ionizing Radiation [1,24]. The results were also compared with results of studies performed by other authors from various countries all over the world [16,17,18,19,20,21,22,23,25,26,27,28,29,30,31,32,33,34,35,36,37,38,39,40,41,42,43,44,45,46,47,48,49,50].

## 2. Materials and Methods

### 2.1. Preparation of Samples for Gamma Spectrometric Measurements

In this study, we examined the concentrations of natural radionuclides ^226^Ra, ^232^Th, and ^40^K in a total number of 60 samples of several cement types, originating from the most popular manufacturers on the Polish market. If the samples were obtained from the same 25 kg bag of cement (e.g., manufacturer X, type CEM-II/A-S 42,5R), they were given the same sample name, e.g., CM-2, etc. The values of obtained concentrations for a given sample name are weighted means of 1–6 values. Particularly, samples CM-1 up to CM-10 are means from four values each, CM-11 to CM-13 are means from six values each, while CM-14 and CM-15 were only examined once (see Table 1).

Cement samples were dried at the temperature of 105 °C for 24 h to obtain a constant sample mass deprived from water, and then sieved to a fraction of 2 mm maximum. Once homogenous materials were procured, they were packed into Marinelli containers with 1.5 dm^3^ volume if measured by MAZAR (or 0.5 dm^3^, if measured by HPGe) and then compacted with the use of a shaker. The containers were filled in such a way that the tested cement remained 5 mm below its top edge. After packing and weighing, the samples were closed and sealed with a tape. The concentration of radium (^226^Ra) was set out on the basis of the activity of its decay products, and with this in mind it was necessary to assure the tightness of the Marinelli beakers to avoid losses in concentrations of radon, which is a volatile decay product of radium (^226^Ra). On the other hand, the concentration of ^232^Th was determined on the basis of measurements of thallium ^208^Tl. For this reason, samples were tested only when the secular equilibrium between ^214^Bi and ^226^Ra and ^208^Tl and ^232^Th has been settled, hence after a period of at least 4 weeks. The 4-week maturing time is sometimes questioned (as too short for settling the secular equilibrium) and in some newest articles the authors use 45-day (or 6-week) time for aging their samples, e.g., [35]. Since the approach to this issue is different in various articles, the authors decided to study cement samples for both 30-day maturing and 45-day maturing, to verify whether there is a statistically significant difference in concentration activity. The values presented in the Results chapter are always a weighted mean of 4 measurements for CM-1 to CM-10 samples, a weighted mean of six measurements for CM-11 to CM-13 and the one value for CM-14 and CM-15 with uncertainty determined by the detector software. In all cases, the statistical deviations from the mean are weighted by the uncertainties of the individual measurements (see the explanation to the Table in Section 3).

### 2.2. Gamma-Rays Spectroscopy

**Figure 2:**
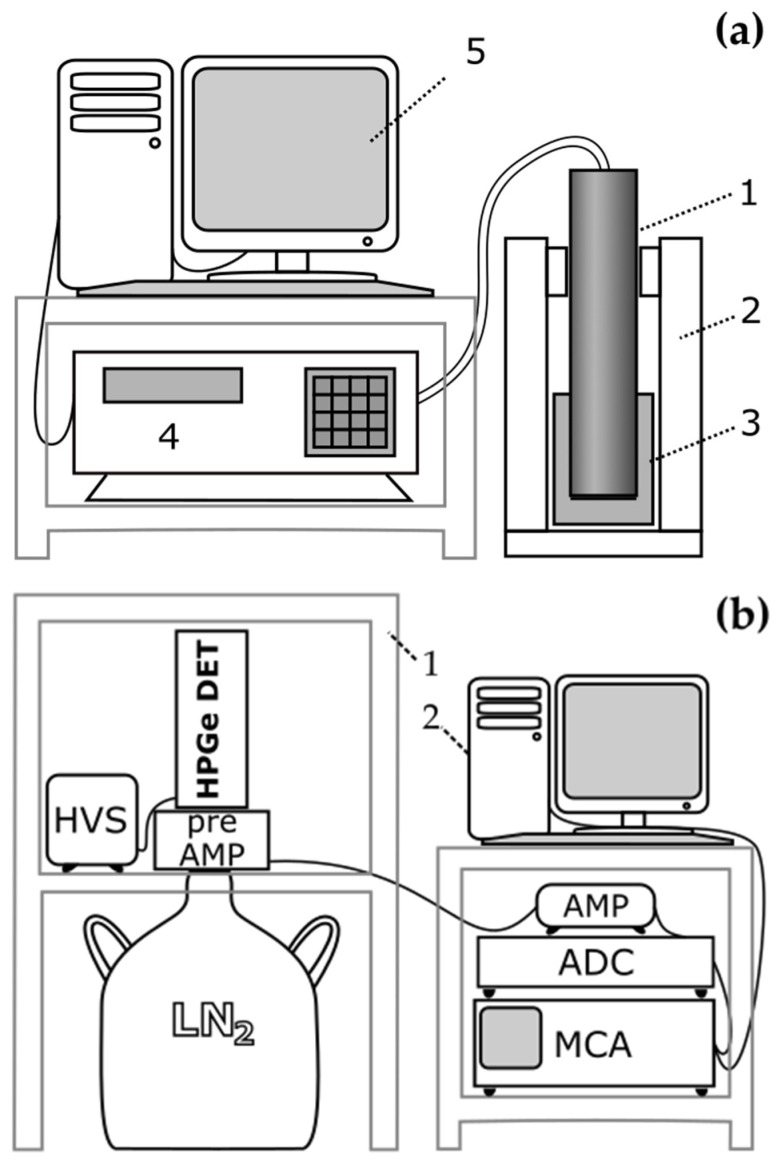
Diagram of experimental apparatus; (**a**) MAZAR: the scintillation probe (1) has been placed in a lead shielding unit (2) with a Marinelli beaker (3) filled with the sample being tested. Its geometry allows pushing the probe (1) into the containers to enable detection of gamma photons from almost the full solid angle. Photons reaching the scintillator are analyzed by the MAZAR spectrometer (4), and the software installed in the PC (MAZAR PC 2007, provided by POLON-IZOT Ltd., Warsaw, Poland), (5) allows observing the spectrum, and calculations of concentrations and radionuclides; (**b**) HPGe: signals from the tested samples are preliminary amplified in a charge-sensitive preamplifier then measured in the LN_2_—cooled germanium detector. The produced electric signal is then amplified in the amplifier, transformed from analog to digital information via AD converter and analyzed in the multi-channel analyzer, then treated in the PC software. Legend: HVS—high-voltage power supply, HPGe DET—germanium detector with a Marinelli container attached, preAMP—preamplifier, LN_2_—liquid nitrogen dewar, AMP—amplifier, ADC—analog-to-digital converter, MCA—multi-channel analyzer, 1—lead cover, 2—PC with GENIE software. For more information, see [53].

In this study, measurements were performed on MAZAR-01 and MAZAR-95 (older model and the newer one of the same spectrometer, described in (a)), and nitrogen-cooled germanium detector HPGe, see (b). Both devices are presented on Figure 2 and described below.

(a) MAZAR spectrometer connected with the scintillation probe NaI(Tl) 2 × 2” (POLON-IZOT Ltd., Warsaw, Poland). The scintillation probe is placed in a lead shielding unit with wall thickness of 50 mm to minimize the radiation background. This is an analyzer that operates in three measurement ranges that allow to determine the radionuclides: ^40^K, ^226^Ra and ^232^Th. Particular measurement channels comprise energy ranges of gamma radiation photons, as follows:
  - channel 1, with energy range of 1.26 MeV–1.65 MeV, detects photons of gamma radiation of the potassium radionuclide (^40^K) with energy of 1.46 MeV, as well as photons from Compton gamma radiation of elements of the thorium and uranium chain, and the apparatus background radiation;
  - channel 2, with energy range of 1.65 MeV–2.30 MeV, detects photons of gamma radiation of the bismuth radionuclide (^214^Bi) with energy of 1.76 MeV, being in secular equilibrium with radium radionuclides (^226^Ra), as well as photons from Compton spectra from the thallium radionuclide ^208^Tl, and the apparatus background radiation;
  - channel 3, with energy range of 2,30 MeV–2.85 MeV, records photons of gamma radiation of thallium (^208^Tl) with energy of 2.62 MeV being in secular equilibrium with thorium (^232^Th) from the thorium chain, and the apparatus background radiation, see e.g., [51,52].
  The detector output calibration has been based on three volumetric measurement calibrations: ^40^K, ^226^Ra, and ^232^Th and the measurement of the matrix of standards as background measurement. Ten calibration coefficients required for setting out radioactive concentrations of potassium ^40^K, radium ^226^Ra, and thorium ^232^Th were calculated with the use of the matrix method. The geometry of the reference sources was similar to that of the tested samples, i.e., it was based on Marinelli beakers of 1.5 dm^3^ volume. The bulk density of reference sources equaled 1.6 g/cm^3^, while the bulk density of samples was contained within the range of 1.1 g/cm^3^ to 1.4 g/cm^3^. To minimize the external background, the detector was placed in a lead shielding unit with wall thickness of 50 mm. The energy resolution of the spectrometer was 6–8%. For every sample, an average value of activity with the uncertainty was calculated. To calibrate the apparatus there were used standard samples: ^40^K (pure potassium chloride KCl, 99.9% b.w.) and ^226^Ra and ^232^Th made on the basis of certified reference materials from the U.S. Department of Energy New Brunswick Laboratory—uranium and thorium ores. The background measurement for the analyzer was made using an aluminum cylinder of weight 1600 g. A detailed diagram of MAZAR apparatus has been presented on Figure 2a.
(b) HPGe of XTRa type (CANBERRA Industries Inc., Meriden, CT, USA). Another equipment used in this study was gamma ray HPGe XTRa detector with a relative efficiency of approx. 30% and 2.0 keV FWHM (at 1332 keV line). The detector, liquid nitrogen cooled, works with a computer equipped with software enabling the calculation of radionuclide concentrations present in the tested sample (GENIE-2000 software, v. 3.2.1, CANBERRA Industries Inc.). The photons energy range of the studied radionuclides lies within the range of from about a dozen to over 2000 keV. The detector is placed in a low-background shielding house, Figure 2b, which ensures a reduction, at least by two orders of magnitude, of the external background of gamma radiation.

Energy and effectivity calibration was performed based on an 80,000 s—measurement of a mixture of 11 isotopes in Marinelli geometry in beakers of 450 mL. The mixture provided calibration in the required energy range and its density was 1.1 g/cm^3^. After taking into account the background of the detector, the radioactive concentration of Bi-214 from several of its power lines was determined. The final value is the weighted average of the radioactive concentrations. The analyzer consists of 8000 channels. For more information on gamma spectrometry via germanium spectrometers, see [53].

### 2.3. Estimation of Radiological Hazard Parameters

Based upon the values of concentrations of natural radioactive radionuclides ^226^Ra, ^232^Th, and ^40^K in the tested cement samples, we were able to set out parameters of radiological hazard to human health. Consequently, to assess the exposure of people to ionizing radiation originating from the above mentioned radionuclides, the following radiological parameters have been set out:

- *Ra_eq_*—radium equivalent activity,
- *H_ex_*—external hazard index,
- *H_in_*—internal hazard index,
- *D*—radiation dose level,
- *E* (AEDE)—annual effective dose,
- *I_γ_*—Gamma radiation activity index,
- *I_α_*—alpha radiation index.

Moreover, the statistical uncertainty estimation was performed for all above-mentioned values, with the use of commonly adopted method of total differential. In our case, based upon this method, one may conclude that since all the listed above parameters *P* are linear combinations of the concentration activities *A_i_*, i.e., $P=\underset{i\in \left\{Ra,Th,K\right\}}{\sum }c_{i}A_{i}$, then their uncertainties must be linear combinations of the uncertainties of the concentrations, Δ*A_i_*, as well, i.e., $\Delta P=\underset{i\in \left\{Ra,Th,K\right\}}{\sum }c_{i}\Delta A_{i}$.

#### 2.3.1. Radium Equivalent Activity

To make comparisons among the materials that contain natural radioactive nuclides ^40^K, ^226^Ra and ^232^Th at different concentrations, the radium equivalent index Ra_eq_ was introduced, that has been set out on the assumption that 10 Bq·kg^−1^ of ^226^Ra, 7 Bq·kg^−1^ of ^232^Th and 130 Bq·kg^−1^ of ^40^K emit the same gamma radiation dose level [16,24,54]. The value of the radium equivalent Ra_eq_ is defined by the following equation:

(1) $$Ra_{eq}\left[{\mathrm{Bq}\;\mathrm{kg}}^{-1}\right]=A_{Ra}+1.43A_{Th}+0.077A_{K},$$

where *A_Ra_*, *A_Th_* and *A_K_* are the concentrations of radium, thorium, and potassium radionuclides, respectively.

For each tested substance the radium equivalent activity should be as low as possible, at most equal to 370 Bq·kg^−1^ to make sure that the gamma radiation dose level is not higher than 1.5 mGy·y^−1^ [54,55]. Should this limit be exceeded, it is considered that the radiation level could be hazardous for human health and life.

#### 2.3.2. Absorbed Gamma Radiation Dose Rate

Another important parameter from the viewpoint of radiological protection is the absorbed gamma dose rate in the air inside a premise. The radiation dose level was calculated applying the conversion coefficients 0.92, 1.1 and 0.08 for radium, thorium, and potassium, respectively [1]:

(2) $$D\left[{\mathrm{nGy}\;h}^{-1}\right]=0.92A_{Ra}+1.1A_{Th}+0.08A_{K}.\;$$

#### 2.3.3. Annual Effective Dose

A parameter closely related to the absorbed gamma radiation dose level is the annual effective dose. Its determination requires the knowledge of the gamma radiation dose level and the value of the conversion coefficient of the absorbed dose in the air into the effective dose. This coefficient is assumed to be a constant value equal to 7·10^−7^ Sv·Gy^−1^. An additional assumption is that, on annual average, humans spend over 80% of their time inside the premises. The equivalent of the annual effective dose is, therefore, calculated on the basis of the following equation [1]:

(3) $$E\left[\mathrm{mSv}\right]=D\left[{\mathrm{nGy}\;h}^{-1}\right]\times 7000\;h\times 7\times 10^{-7}{\mathrm{Sv}\;\mathrm{Gy}}^{-1}.$$

The value of the annual effective dose recommended by UNSCEAR (2000) should not exceed 1.0 mSv during a year to be able to consider a building material as safe with respect to radioactivity for human health.

#### 2.3.4. External Hazard Index

Further two parameters, namely the external and internal hazard indices, have been defined by Beretka and Mathew [16], and describe the hazard connected with internal and external radiation. The external hazard index can be obtained from the expression of Ra_eq_, based on the assumption that its admissible maximum equal to 1.0 conforms to the upper limit of the radium concentration equivalent Ra_eq_ (370 Bq·kg^−1^). The external hazard index (*H_ex_*) is therefore expressed as follows:

(4) $$H_{ex}=\frac{A_{Ra}}{370}+\frac{A_{Th}}{259}+\frac{A_{K}}{4810}\;$$

and is dimensionless.

#### 2.3.5. Internal Hazard Index

Apart from the external hazard index, originating from ^40^K, ^226^Ra, ^232^Th, equally important is exposure to the internal radiation coming from radon ^222^Rn and its short-lived decay products, which are particularly hazardous for the respiratory tract (primarily for the bronchi and lungs). The internal hazard index is set out based on the following equation:

(5) $$H_{in}=\frac{A_{Ra}}{185}+\frac{A_{Th}}{259}+\frac{A_{K}}{4810}$$

and is dimensionless, as well.

The value of the internal hazard index must be lower or at least equal to one, so that the hazard of radiation originating from radon and its decay products remains insignificant and negligible.

#### 2.3.6. Gamma Radiation Activity Index

To verify whether the dose criterion for materials commonly used in the construction sector has been met, the gamma radiation activity index was set out in accordance with the following equation [4]:

(6) $$I_{\gamma }=\frac{A_{Ra}}{300}+\frac{A_{Th}}{200}+\frac{A_{K}}{3000}.$$

The gamma activity index takes into consideration the method and amount of the given type of material used on construction. Limiting values of the gamma radiation activity index, which should not be exceeded by materials, have been listed in Table 2.

If *I_γ_* ≤ 1.0, the annual effective dose is lower or at least equal to 1 mSv for construction materials used in large quantities. On the other hand, *I_γ_* ≤ 0.5 conforms to an annual effective dose lower or at least equal to 0.3 mSv if the construction material is used in bulk. Similarly, if *I_γ_* ≤ 6.0, then it conforms to an annual effective dose lower or equal to 1.0 mSv if the material is used outside a premise. Furthermore, when *I_γ_* ≤ 2.0, this conforms to an annual effective dose lower or equal to 0.3 mSv if the material is used outside a premise.

#### 2.3.7. Alpha Radiation Index

In addition, the alpha radiation index has been set out, which defines exposure to alpha radiation coming from radon and its short-lived decay products present in building materials. This parameter has been defined as [24,56]:

(7) $$I_{\alpha }=\frac{A_{Ra}}{200}.$$

The International Commission on Radiation Protection recommends that the radon concentration in closed premises remains lower than 200 Bq·m^−3^. Supposing that in the sample the radium concentration is assumed to be at the maximum admissible value equal to 200 Bq·kg^−1^, then the value of the alpha radiation coefficient equals one. If the concentration of radium radionuclide in construction materials is higher than 200 Bq·kg^−1^, the concentration of radon inside the premise may exceed the admissible value of 200 Bq·m^−3^, which is considered to be safe for humans (and which is set up by the International Commission on Radiation Protection) [24,40,56]. On the other hand, it is also assumed that if the concentration of the radium radionuclide (^226^Ra) is no higher than 100 Bq·kg^−1^, the concentration of radon inside the premise should be lower than 200 Bq·m^−3^.

### 2.4. Statistical Methods

A further objective of the paper was to conduct a broad statistical analysis aimed at verifying dependencies and correlations between particular concentrations of radionuclides. With this in mind, first, it was verified whether there were outliers in any of the trials, and whether the concentration of a given radioactive element was normally distributed. The answer to the first question may be obtained, for example, using box-plots, and to the second one—by executing the Shapiro–Wilk test (for introductory information see [57,58,59,60,61]). Just as any statistical test, this particular test is used to verify the null hypothesis on the accepted significance level of (1 − α) × 100%,which in the case of the Shapiro–Wilk test is the normality of distribution, i.e., whether it may be presumed that the given trial is the one with normal distribution. Similarly, as in each statistical test, an evaluation is made whether the put null hypothesis should be rejected in favor of the alternative hypothesis, or whether it is impossible to reject it. In the majority of statistical programs, the adopted null hypothesis is that the given trial has the normal distribution. If the *p* value is higher than the assumed significance level α, the null hypothesis cannot be rejected; if it is lower—it should be rejected in favor of the alternative hypothesis on distribution non normality [59,60]. Since only the set of concentrations of radionuclide ^40^K has positively passed the Shapiro–Wilk test, the further statistical analysis needed to be based on the Wilcoxon–Mann–Whitney non-parametric tests as a counterpart of the Pearson test for correlation and the Kruskal–Wallis test as a counterpart of the one-way ANOVA for needs of determination of dependencies between the concentrations. In addition, the correlation coefficient in trial, ρ, and 90% confidence interval for ρ were determined. In regards to the correlation analysis, please refer to the study in [62]; a good introduction to the one-way ANOVA one can find in the lectures available on the webpage of the Faculty of Genome Science of the University of Washington [63], and about non-parametric tests—see [64]. All calculations and tests, as well as diagrams, have been executed in R program, i.e., an open-source software for data analysis, the full documentation of which is available in [65].

## 3. Results

### 3.1. Natural Activity Concentration

Table 3 lists the concentrations of natural radioactive radionuclides ^226^Ra, ^232^Th, and ^40^K of the tested cement samples, and their average concentration has been set out. Both arithmetic and weighted means were calculated, while their uncertainties were estimated with the use of the standard deviation and average weighted deviation, respectively. When calculating the weighted deviation, the weights $w_{i}={\left(\frac{1}{\Delta x_{i}}\right)}^{2}$ were used, where $\Delta x_{i}$ is the uncertainty determined for every of the values $x_{i}$, hence the relatively lower uncertainty values for the weighted mean. The weighted mean itself is higher than the normal arithmetic mean since higher values entered the weighted mean with higher uncertainties (both values are provided in Table 3, for comparison). However, it should be kept in mind that properly one should calculate the weighted average if every value in the sample has a different uncertainty.

The distribution of radionuclides ^40^K, ^226^Ra and ^232^Th is not homogenous in the tested cement samples. As may be seen from data presented in Table 3, concentrations of radionuclides of potassium, radium and thorium in cement samples remain within the range of 123–430 Bq·kg^−1^, 21.7–75.7 Bq·kg^−1^, 12.3–47.3 Bq·kg^−1^, with the average of 283 Bq·kg-^1^, 48 Bq·kg^−1^ and 29 Bq·kg^−1^ respectively. Average global values of the concertation of potassium, radium, and thorium in the Earth’s crust presented in the UNSCEAR Report (2000) equal 400 Bq·kg^−1^, 35 Bq·kg^−1^, and 30 Bq·kg^−1^, respectively [1].

These results allow the presumption that, only in the case of the potassium radionuclide, its mean concentrations in cement samples are by about 1/3 lower than the mean contents of this radionuclide in the Earth’s crust. Meanwhile, the average concentrations of the radium radionuclide in the tested cement samples are higher than the average concentration of that radionuclide in the Earth’s crust. Moreover, in most of the samples the concentration of ^226^Ra reaches or even exceeds the recommended 35 Bq/kg limit (only those for CM-1, CM-5, CM-11, and CM-14 are below the limit). On the other hand, the average concentration of the thorium radionuclide in tested cements assumes a value close to the mean content of that radionuclide in the Earth’s crust. Nonetheless, attention should be drawn to the fact that in six samples this value had been exceeded (within the statistical error) i.e., CM-3, CM-7, CM-8, CM-12, CM-13, and CM-15.

Such a broad dispersion of concentration values of natural radioactive radionuclides in cement arises, in the first place, from different type of raw materials used in production of cement and the contents of other additives, such as volatile ashes, slags, the contents of which may also comprise natural radioactive radionuclides. These values could also be influenced by time- and composition-dependent co-combustion of waste products. Published research indicates that the addition of volatile ash to construction materials may cause increased concentrations of the ^226^Ra radionuclide, which is also visible in the tested cement in this study. The lowest concentrations of natural radioactive radionuclides were obtained for Portland cement containing no more than 0–5% of secondary materials, i.e., for samples CM-1, CM-4, CM-5, CM-11, and CM-14. The highest concentrations of natural radioactive radionuclides of potassium, radium and thorium were obtained for samples of pozzolan cement, i.e., CM-3, CM-8 and CM-15, but also samples of CM-2, CM-3, CM-6, CM-7 and CM-10, CM-12, and CM-13 which are Portland cements with an additive of slag fume or fly ash CEM-II. The pozzolan cement contains the highest concentrations of additives, and namely 36–55% of silica fume and natural and industrial pozzolan and silica volatile ash, as well as 0–5% of secondary ingredients.

Additionally, Table 4 presents the mean concentrations of natural radionuclides of radium, thorium and potassium in the tested samples coming from other countries. Each country has been denoted by the ISO 3166–1 alpha-3 code. Most of the data from other studies were determined without uncertainty, which made it impossible to accurately determine both the weighted mean, and the weighted deviation, as is required for proper procedure. Therefore, in Table 4, all data arithmetic mean and standard error of the mean (abbr. SEM) were calculated instead. The weighted mean and weighted deviation for all activity concentrations also have been calculated; however, this is on the basis of data from Albania, Greece, Pakistan, Senegal, and Turkey only.

Data specified in Table 4 allow a presumption to be made that much higher concentrations of the radium radionuclide in relation to Polish cements have been obtained for samples of cement coming from Greece, Senegal, Malaysia, India, and Turkey. An exceptionally high value was achieved in samples from Turkey, were activity concentration of ^226^Ra was even more than nine times of the average content in the Earth’s crust. On the other hand, in the remaining countries concentrations of radium radionuclide in cement were comparable or lower as compared to the tested material samples. Given the mean concentrations of the thorium radionuclide, it may be assumed that much higher concentrations of this radionuclide are present in samples coming from Australia, Brazil, Egypt, Italy, Malaysia, and Turkey, as compared to the tested cements. As for the remaining samples of cement originating from other countries’ markets, the concentrations of the thorium radionuclide were comparable or lower in relation to tested cement samples in the present study. A comparison of concentrations of the potassium radionuclide of samples of Polish cements with samples of cements coming from other countries allows the presumption that only for cement samples coming from Brazil, Cuba, India, Slovakia, Turkey, and Yemen the highest concentrations of that radionuclide were recorded. Regarding the remaining countries, concentrations of the potassium radionuclide were comparable or lower as compared to the tested samples.

### 3.2. Statistical Analysis

**Figure 3:**
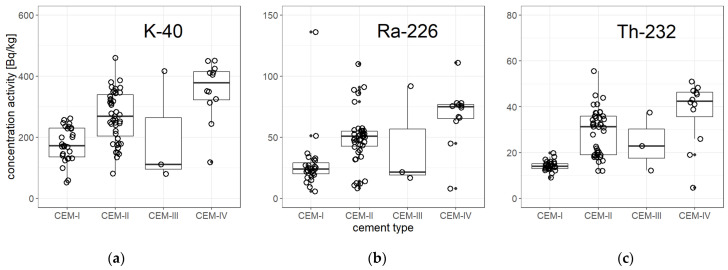
Box-whisker plots for the concentration of potassium (**a**), radium (**b**), and thorium (**c**). Box-plots present the basic values of descriptive statistics: the median (horizontal line), the first and the third quantile (bottom and top side of the box, respectively), the maximum and minimum values not considered an outlier (upper and lower whisker border, respectively), and outliers and extreme values. The figure contains separate boxes for different cement types as to extract statistical differences between these groups, if exist. All the collected data, both from other studies and the measurements of the authors of this study, were used to prepare the above charts.

**Figure 4:**
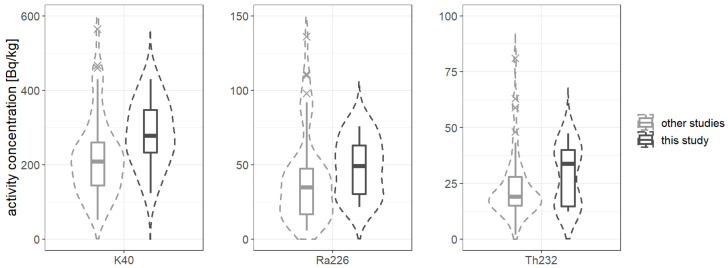
The violin-type plots of activity concentration of ^40^K, ^226^Ra and ^232^Th. Data are grouped into “other studies” (left light-gray graphs) and “this study” (right dark-gray graphs). The violin-type plots are in fact a type of density plots and show how the measurements are distributed through the sample. Strong asymmetry is clearly visible in all plots for data from the other studies. Measurements performed by the authors reveal quite low asymmetry or even symmetry for ^40^K concentration.

From the statistical point of view, it should be determined the density distribution of the concentration activity for each radionuclide and verified whether they are normal distributions or not. In addition, it should be determined whether there exist statistical differences between concentration activities within cement groups: CEM-I, CEM-II, CEM-III, CEM-IV, and CEM-V. At last, the goal is to find whether any dependencies exist between concentrations for different types of cements and correlations between particular concentrations. Therefore, the first step is to perform some basic descriptive statistics and normality tests. For this purpose, Shapiro–Wilk tests were executed, also box-whisker, histograms and violin plots were performed, see Figure 3 and Figure 4. Within cement types: CEM-I, CEM-II, CEM-III, and CEM-IV, for none of the features: concentration of ^40^K, concentration of ^226^Ra, and concentration of ^232^Th extreme values have been recorded. Only in the CEM-V group were there extremely high values of all concentrations. However, this group was also low-represented, so statistically insignificant, it was omitted on the figures, but accounted in further discussion. It should be emphasized that all distributions have outlier values, moreover, they indicate strong asymmetry, which is clearly proven by calculating skewness coefficients and verifying statistical tests for normality. Indeed, the strong lack of symmetry of the distributions has been confirmed by rejection of the null hypothesis in the Shapiro–Wilk tests, see also Table 5, which summarizes all statistical analysis performed in this section.

The Shapiro–Wilk test is used to assess, on the given significance level α, whether one may assume that the given group of measurements is a feature with normal distribution or not, by calculating the so-called *p* value. If *p* > α, the hypothesis of normality of the distribution cannot be rejected; if *p* < α, then the null hypothesis should be rejected in favor of alternative hypothesis that the distribution is not normal. In such a case further work with the group is based on non-parametric tests. It was calculated for potassium *p* = 0.06, radium *p* = 1.5 × 10^−5^ and thorium *p* = 2 × 10^−6^; thus, only for ^40^K the null hypothesis could not have been rejected, but the result is doubtful, as *p* is very close to α. Moreover, Figure 4 shows that concentrations of radium and thorium are characterized by strong right-side asymmetry (long high-values tails), especially in the group of data from other studies (light-gray graph) which is confirmed by the calculation of the skewness coefficient, presented together with other statistical parameters in Table 5. Data obtained by the authors of this article present lower asymmetry (dark gray graph) which may indicate a more homogeneous cement market in Poland.

This asymmetry in distribution of the activity concentration is puzzling, especially that there is a “long tail” for large contents of both radionuclides, ^226^Ra and ^232^Th. As the sample size is relatively high (it amounts 60 results from our study and 63 from other works, which comes up to a total of 123), the preliminary conclusions may be drawn. It seems that the tails result from the application of large amounts of cements with dusts (i.e., the so-called grey dusts), which have been subject of numerous published studies. As it was emphasized earlier in the article, the non-normality necessitates the use of non-parametric statistical tests in further statistical analysis.

**Figure 5:**
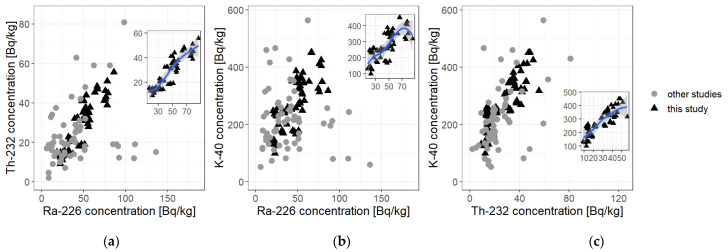
Diagram of dependencies of the concentration activity of thorium ^232^Th on radium ^226^Ra (**a**), potassium ^40^K on radium ^226^Ra (**b**), and potassium ^40^K on thorium ^232^Th (**c**). Quite strong correlation is clearly visible on all of the plots; however, the strongest one is for Ra-Th dependence. A linear dependence with a narrow confidence corridor at the level of 99% is only noticeable in the pair of Ra-Th for data obtained by the authors, and a considerable dispersion of values and a wide confidence corridor are noticeable in two remaining cases, and prove a weak dependence in the correlation, though it still remains positive.

With an available statistical base for all the three trials one may pass on to an analysis of binary setups of ^226^Ra—^40^K, ^226^Ra—^232^Th, and ^232^Th—^40^K. Figure 5 presents dependence diagrams for each of those setups, respectively, devised for all results coming from studies performed by other authors (light-gray circles) and from this study (black triangles).

The Kendall non-parametric test for correlation has been carried out for all the binary systems and the correlation coefficient in trial and 95% confidence interval for ρ have been determined. For this test, in all cases the *p* value was found to be extremely low, even of the order of 10^−16^ to 10^−6^, thus much lower than the significance level α, which suggests that it is impossible to reject the hypothesis of a dependence between the concentrations of ^40^K, ^226^Ra, and ^232^Th.

The non-parametric equivalents of a single-factor ANOVA analysis, namely the Kruskall–Wallis and pairwise WMW tests, were also implemented, which provide the answer to the co-dependence problem for groups within the samples. The authors’ interest was whether there exists a significant difference between cement types and also between data obtained for different aging time, especially to draw conclusions if there exists a statistical difference in activity concentrations between cement samples, which were shorter aged (30 d) and longer aged (45 d). The results of the tests together with discussion have been moved to the Discussion section.

What is especially noticeable is that measurements of the radioactivity concentration are not normally distributed, and in some cases non-normality is extreme (^226^Ra for “all data” and ^232^Th for “all data”, as well). This issue will be discussed further. Additionally, in all cases *p* value for Kendall rank test is substantially lesser than the adopted significance level α. This means that there exist correlations within the binary groups; K-Ra, Ra-Th, and K-Th. The conclusion is supported by the calculated 90% confidence level for the correlation coefficient which in all mentioned cases is above 0, though for K-Ra pair, the lower limit exceeds 0 only slightly. The highest correlation is between the concentration of radium and thorium radionuclides; however, in all cases the strong relationship is clearly visible, see also Figure 5.

### 3.3. Evaluation of Radiological Threats

Table 6 presents values of radiological parameters calculated on the basis of Equations (1)–(7). Concentrations of the radium equivalent activity for the tested cements remained within the range of 48.4–173 Bq·kg^−1^, as shown in column two of Table 6. The obtained results show that the lowest *Ra_eq_* values were obtained for Portland cements CM-1, CM-4, CM-5, CM-11, and CMT-14. On the other hand, the highest *Ra_eq_* value was recorded for samples of pozzolan cement, i.e., CM-3, CM-8, and CMT-15, and for two samples of Portland cement of CEM-II type with high contents of ash, namely CM-2 and CM-7. The broad range of *Ra_eq_* values suggests that continuous monitoring of the radioactivity level of new types of cement, before using them as a building material, should be provided. To be able to consider that the given cement/material is safe with respect to radioactivity, the *Ra_eq_* value needs to be lower or at most equal to 370 Bq·kg^−1^ to make sure that the value of dose rate remains not higher than 1.5 mGy·y^−1^ [16,55]. For all the tested cements the value of radium equivalent was found to be much lower than 370 Bq·kg^−1^, which suggests that the usage of those materials in construction may be considered as safe from the viewpoint of radiological protection.

The next calculated radiological hazard parameter is the rate of the dose absorbed in the air inside a premise, and its values for particular samples are included in the third column of Table 6. The average range of the dose rate remained between 43.1–153.8 nGy·h^−1^. Average D values for eight samples of cements (CM-2, CM-3, CM-6, CM-7, CM-8, CM-12, CM-13, and CM-15) were higher than the average global gamma radiation dose level inside a premise, i.e., 84 nGy·h^−1^ [1]. The highest D value was obtained for samples CM-3, CM-8, and CM-15, which all are pozzolan cements, and their values of absorbed dose rate are 70–80% higher than the global average of gamma radiation dose level inside a premise.

**Figure 6:**
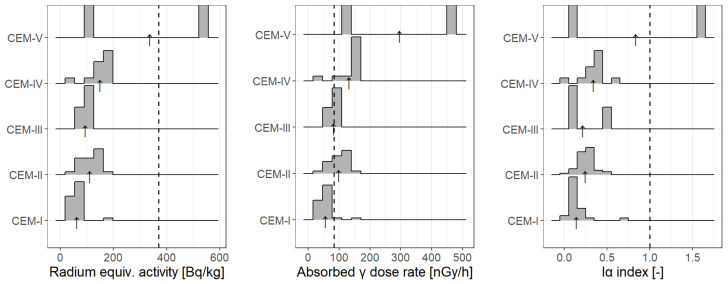
Histogram graphs for hazard indices *Ra_eq_*, *D* and *I_α_* for five types of cements, based on such data from Table 4, for which the authors provided information on the type of cement. The arrows indicate the weighted averages within the group.

Table 6 includes values obtained from the authors’ own data. However, for a wider analysis, data from various studies were compiled, and Figure 6 shows the parameters *Ra_eq_*, *D* and *I_α_* calculated from these data. They are collected in a form of density plots and grouped by cement types: CEM-I, CEM-II, CEM-III, CEM-IV, and CEM-V. Very few data have been found in the literature for other types of cements than Portland, pozzolan and Portland composite cement, namely 3 for CEM-III and 2 for CEM-V. Nevertheless, they are presented here to indicate the need for such an analysis, especially for CEM-V, where in one of the samples the values of the calculated radiological parameters were extremely higher than the established limits. Except this one case, all other values did not exceed the limits (which are indicated on Figure 6 as dashed lines).

On the other hand, mean values of *I_α_* for the tested cement types were presented in column eight of Table 6. The maximum *I_α_* value was recorded for the cement samples CM-3 (0,378), CM-8 (0,368), and CM-15 (0,379), while the lowest one for samples CM-11 (0.108), CM-1 (0.124), CM-5 (0.125), and CM-14 (0.135). In none of the cement samples was the concentration of radionuclide ^226^Ra over 200 Bq·kg^−1^ detected. The highest concentration of this radionuclide was obtained for samples CM-3 and CM-15, both pozzolan cements, and they equaled 75.6 and 75,7 Bq·kg^−1^, respectively (see Table 3), hence the index *I_α_* equaling almost 0.38 in both cases. The obtained results allow the presumption that the concentration of radon inside a premise, in which the tested cements are used, would be lower than 200 Bq·m^−3^.

Calculated values of the annual effective dose were presented in the fourth column of Table 6. The average values of the annual effective dose remain within the range of 0.21–0.75 mSv. Data presented in the report of UNSCEAR (2000) suggest that, in the whole world, persons staying inside a premise built from construction materials receive an effective dose at the level of 0.4 mSv yearly [1]. This suggests that mean E values for eight samples of cements (again, they are: CM-2, CM-3, CM-6, CM-7, CM-8, CM-12, CM-13, and CM-15) were higher than the value of 0.4 mSv. The highest E value was obtained for two samples, CM-8 and CM-15 (both Pozzolan). Nevertheless, for none of the tested cement samples the value 1.0 mSv/y has been found to be exceeded. Consequently, it may be considered that the tested materials are safe for human health and may be used in construction.

**Figure 7:**
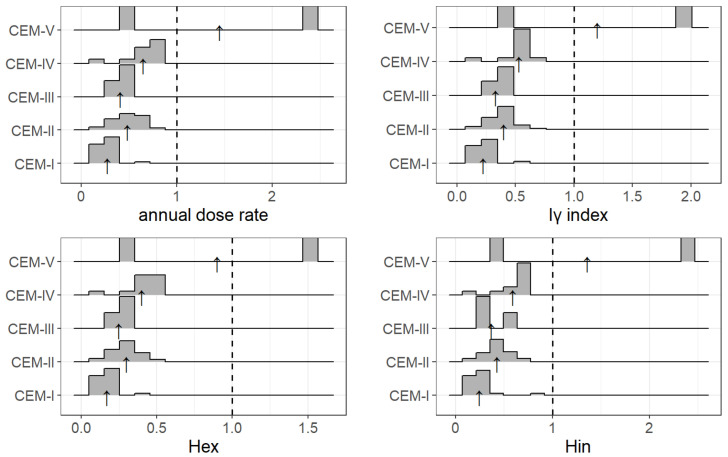
Histogram graphs for hazard indices *E*, *H_ex_*, *H_in_*, and *I_γ_* for five types of cements, based on such data from Table 4, for which the authors provided information on the type of cement. The arrows indicate the weighted averages within the groups.

Mean values of external hazard indices for the tested cement samples have been presented in column five of Table 6. The calculated values of *H_ex_* remain within the range of 0.13–0.47. On the basis of obtained results, the presumption may be made that the lowest values, *H_ex_*, were recorded for Portland cement: CM-1, CM-4, CM-5, CM-11, and CM-14. On the other hand, the highest value *H_ex_* was obtained for samples of pozzolan cement, i.e., CM-3, CM-8, and CM-15, and for one sample of Portland cement with the addition of slag ash CM-7. For all the tested samples of cement the *H_ex_* values were lower than one, and hence it may be presumed that the tested materials are safe with view to natural radioactivity. The distribution of *H_ex_* values has also been presented as a diagram on Figure 7, where results have been grouped by the type of cement CEM-I, CEM-II, CEM-III, CEM-IV, and CEM-V for all collected data, also from other studies, where there were data possible to retrieve relevant information on the cement type.

Average values of internal hazard indices for the tested cement samples were presented in column six of Table 6. The calculated *H_in_* values remain within the range of 0.19–0.673. Again, the highest *H_in_* values were recorded for samples of pozzolan cement (CM-3, CM-8, CM-15) and Portland slag or fly-ash cements (CM-2, CM-7, CM-12), while the lowest values were reached for samples CM-1, CM-4, CM-5, and CM-11, i.e., the Portland ones. Furthermore, Figure 7 presents values of *H_ex_* and *H_in_* grouped appropriately for different types of cements CEM-I (Portland cement), CEM-II (Portland composite cement), CEM-III (blast-furnace metallurgic Portland cement), CEM-IV (pozzolan cement), and CEM-V (composite cement being a mixture of Portland, slag and fly-ash cement, and pozzolana). For each of the groups a histogram plot has been carried out that presents the distribution of the values of given parameter within each group (the arrows indicate the means, Figure 7). It is clear that on average the highest internal hazard index was recorded for pozzolan cement, and the lowest one for Portland cement. Nevertheless, for all the cement samples subjected to testing the values *H_in_* were lower than 1, therefore they may be considered safe with respect to natural radioactivity.

Average values of gamma radiation activity for the tested cements were shown in the seventh column of the Table 6. Values of *I_γ_* remain within the range of 0.174–0.624. The lowest *I_γ_* values were obtained for samples of Portland cement, i.e., CM-1, CM-4, CM-5, CM-11, and CM-14. On the other hand, the highest *I_γ_* values were obtained for samples of pozzolanic cements (CM-3, CM-8, and CM-15), and for slag or fly-ash cements (CM-2 and CM-7). As regards to materials used in large quantities, such as cement, *I_γ_* has to be lower or at most equal to 1, so that the annual effective dose does not exceed the level of 1 mSv. Calculated values of *I_γ_* for the cement samples measured in this study were indeed lower than one, therefore their usage is safe from the radiological protection point of view. Data for *I_γ_* have also been graphically presented on Figure 7.

## 4. Discussion

### 4.1. General Discussion

The paper presents the concentrations of natural radioactive isotopes of radium (^226^Ra), thorium (^232^Th) and potassium (^40^K) for various types of cements commonly used in the construction industry. These are cements commercially available in Poland. The main aim of this article was to show the problem of natural radioactivity of cement. The radioactivity of concrete as the final product obtained from the combination of coarse and fine aggregate and a binder consisting of a mixture of water with cement and additives, was not investigated on purpose. Such tests are planned after detailed determinations of individual concrete components are made. Aggregate constitutes approx. 70–80% of the total volume of concrete and has a significant impact on the characteristics of both fresh concrete mix and hardened concrete. Aggregate of different fractions, i.e., different grain sizes, should be used. Most often, fine aggregate is used—sand, and coarse—gravel. Earlier studies of the authors showed that granite aggregate slightly exceeds the natural radioactivity in comparison with other commonly used ones. The authors of the article notice a number of advantages of granite aggregates. These advantages are presented in numerous publications. As granites are becoming more and more popular as building materials, the measurement of the concentrations of natural radioactive isotopes is also particularly important in their case, due to the assessment of human exposure to ionizing radiation emitted by them. It should be stated here that all other aggregates are characterized by natural radiation at diverse levels.

As for the results of this study, first of all, presented data show that statistically (by average) none of the tested cement types exceeded the values accepted as safe for human life in the context of radiological protection, though some individuals did. The results stay in accordance with most of other studies. Undeniably, in some cases the activity concentrations were extremely high, but they were very rare cases, and bad statistics (low sample size). This is why, in the authors’ opinion, it would be much preferred to perform more measurements on cement types which were low-represented in the cited articles, e.g., CEM-III (3 reported cases) and especially CEM-V cement types (2 reported cases). With more data, one would be able to perform even better statistical analysis, especially to verify dependencies between mean concentrations in each cement type and/or diversity between the groups. Moreover, there would be a better chance of checking the normality of the statistical samples in each group; for now—the samples are not normal, so non-parametric tests have to be performed. This is not a great difficulty, but the non-normality is indeed puzzling as there is no candidate for the genesis or explanation for this state. Nonetheless, based on the data collected for the purpose of this paper, several conclusions to be discussed emerged:

(1) Portland cements are these of lowest (by average) activity concentrations of ^40^K, ^226^Ra and ^232^Th;
(2) There is a need to collect data with more additional information, including the types of studied cement, to verify variability between various groups;
(3) Results from this study suggest that the highest by average activity concentration one should expect in CEM-IV cements. However more data is needed to verify whether these values for CEM-V are even higher or not; vide Figure 6 and Figure 7;
(4) Kruskal–Wallis test, which is a non-parametric version of one-way ANOVA (i.e., compares means between various groups), proves, on 95% significance level, that there are significant differences of activity concentrations within groups (cement types), see Table 7;
(5) Pairwise WMW tests comparing the data within cement types’ groups reveal that, at 95% significance level, one can postulate that:
  - the lowest concentrations of the natural radionuclides are in CEM-I type of cements, i.e., Portland;
  - CEM-II (slag, fly-ash or composite cements) possess higher activities of the radionuclides than in Portland;
  - the highest values are observed in CEM-IV, see Table 7.
    As it is easily visible, in all cases (activities of ^40^K, ^226^Ra, ^232^Th for all data or for this study data only) the *p* value is significantly less than α = 0.05.
    Pairwise WMW tests were performed for groups “CEM-I”, “CEM-II”, “CEM-III”, and “CEM-IV” cement types.
(6) The question is whether the quite high correlation (determined for all collected data) should be in a way explained; is it a very nature or a chance because of relatively small size (n = 120)? The authors tend to conclude that this is not a random effect, but augmented data would give more reliable answer and could reveal some dependencies which at this moment are merely supposed. To clarify this, let us have a look again at Figure 5. In all cases (Ra-Th, Ra-K, and Th-K), there is a main branch of a more or less linear relationship, but there are a few data lying beneath (high concentration of radium, low concentration of the other radionuclides). These values may be due to several properties. First one is the geological origin, the second one—production technology. One should notice that among the sparse data of small ^40^K concentration and high ^226^Ra concentration are those from Senegal [35], and samples are of CEM-II, CEM-III, and CEM-IV types, thus with fly ash or slag or other materials, which may possess specific geological origin or specific production technology. The above-mentioned main branch reflects the almost-linear relationship: the higher radium concentration, the higher potassium and thorium concentration. Some data, however, elude this pattern and more data would give a better inference.

**Table 7:** Results of Kruskal–Wallis and pairwise Wilcoxon–Mann–Whitney tests which, at a given significance level, compare means of the features within given groups. In this article the features are activity concentrations of the radionuclides, first for all collected data from Table 4 for which the information on cement type was provided, next for the authors’ own data only. The groups, in turn, are cement types.

Statistics	^40^K —All Data	^40^K —This Study	^226^Ra —All Data	^226^Ra —This Study	^232^Th —All Data	^232^Th —This Study
Kruskal-Wallis Test	*p* = 0.220 > α	*p* = 0.355 > α	*p* = 0.162 > α	*p* = 0.380 > α	*p* = 0.110 > α	*p* = 0.228 > α
Pairwise WMW test for cement types ^(1)^	CEM-II > CEM-I (*p* ~ 10−5)	CEM-II > CEM-I (*p* ~ 10−6)	CEM-II > CEM-I (*p* ~ 10−6)	CEM-II > CEM-I (*p* ~ 10−8)	CEM-II > CEM-I (*p* ~ 10−9),	CEM-II > CEM-I (*p* ~ 10−9),
	CEM-IV > CEM-I (*p* ~ 10−5)	CEM-IV > CEM-I (*p* ~ 10−7)	CEM-IV > CEM-I (*p* ~ 10−4)	CEM-IV > CEM-I (*p* ~ 10−5)	CEM-IV > CEM-I (*p* ~ 10−4)	CEM-IV > CEM-I (*p* ~ 10−5)
	CEM-IV > CEM-II (*p* ~ 10−3)	CEM-IV > CEM-II (*p* ~ 10−4)	CEM-IV > CEM-II (*p* ~ 10−3)	CEM-IV > CEM-II (*p* ~ 10−5)	CEM-IV > CEM-II (*p* = 0.01)	CEM-IV > CEM-II (*p* ~ 10−5)

^(1)^ “Cement types” is the grouping variable; one-side test, the alternative hypothesis is for being greater.

### 4.2. NORMS

The majority of radionuclides in NORM arise from uranium and thorium decay. Lots of operations and activities induce concentration and redistribution of NORMs in the environment, wherein the major contributors come from mining and minerals processing facilities (e.g., uranium mining and milling, copper/gold/tin/iron/lead/coal/granite/limestone mines, phosphate rock mining, and production of phosphoric acid) and industrial processes, among which the most important are operations producing building materials from mine wastes (phosphogypsum and phosphate slag), fertilizer production utilizing phosphoric acid, operations using fly ash from coal mines, the manufacture of catalysts and special glasses from rare earths etc. [68]. Many researchers all over the world study the natural radioactivity of these materials; therefore, the literature on this topic is very rich. For example, publications of the group of researchers gathered under the Cost Action-Norm. They provide extensive database which is formed as a tool for radiological assessment of building materials [69,70,71,72,73].

### 4.3. Aging Time

**Figure 8:**
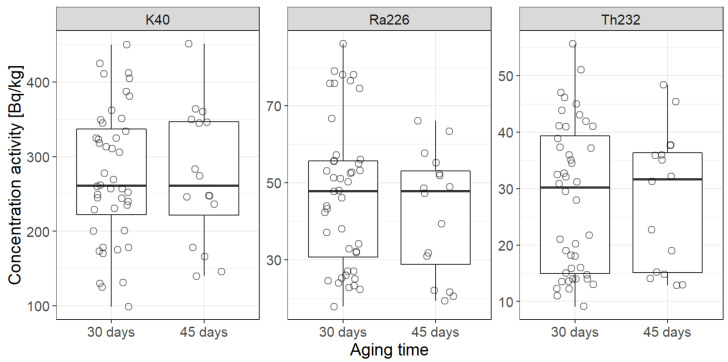
Comparison of activity concentration of the natural radionuclides in the case of 30-d aging vs. 45-d aging. The comparison is made with the use of box-whisker plots. Medians in all cases are almost equal.

**Figure 9:**
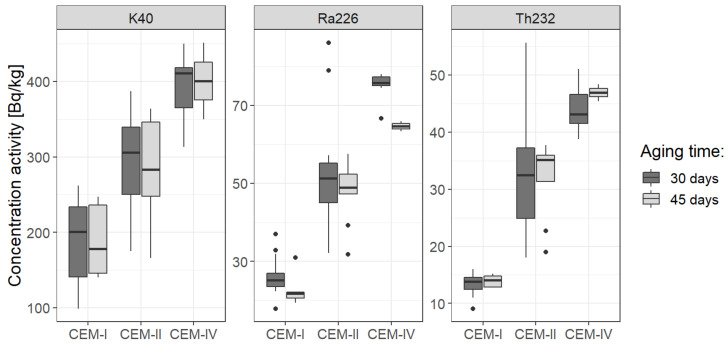
Comparison of activity concentration of the natural radionuclides in the case of 30-d aging vs. 45-d aging in the three types of cement: CEM-I, CEM-II, and CEM-IV. The comparison is again made with the use of box-whisker plots. This time, medians are not always the same, see discussion.

The secular equilibrium is achieved when activities of the parent and daughter nuclides become equal. In the case of ^226^Ra and ^222^Rn, based on simple calculations form the decay law, at least seven times the half-time of radon is sufficient, hence 7 × 3.8 d ≈ 28 d. However, in many studies the researchers seal the samples for about 6 weeks (or 42–45 days), so it looks interesting whether there is a statistically significant difference between activity concentrations in both aging times applied, namely 30 days and 45 days. For potassium-40 being one of the primordial radionuclides, whose half-time equals 1.26 million years, there is no difference between 30 and 45 days, indeed, and this fact is also reflected in statistical analysis, summarized in Table 8 and Figure 8 and Figure 9. Therefore, WMW test (with the use of Hochberg method) was applied for two groups with samples sealed for 30 days and for 45 days.

**Table 8:** Descriptive statistics and results of statistical tests performed to verify whether there exists a statistically significant difference between activity of ^40^K, ^226^Ra, and ^232^Th in the cases of shorter and longer aging time. WMW tests have been conducted with no division into groups of various cement types, while weighted means with weighted deviations have been calculated within these groups.

Statistics	Cement Types		^40^K Activity	^226^Ra Activity	^232^Th Activity
WMW Test for Aging Time 30 d vs. 45 d ^(1)^	All		Statistically Indistinguishable (*p* = 0.51)	Statistically Indistinguishable (*p* = 0.19)	Statistically Indistinguishable (*p* = 0.56)
Weighted mean ± weighted deviation in particular groups	CEM-I	30 d (n = 14) ^(2)^	197 ± 52	26.3 ± 5.0	13.6 ± 1.8
		45 d (n = 5)	197 ± 46	22.0 ± 4.1	14.1 ± 1.3
	CEM-II	30 d (n = 23)	297 ± 60	53 ± 13	33.8 ± 9.6
		45 d (n = 9)	304 ± 58	52.4 ± 5.6	32.9 ± 6.0
	CEM-IV	30 d (n = 7)	393 ± 49	75.0 ± 3.7	44.5 ± 4.0
		45 d (n = 2)	408 ± 52	64.7 ± 2.1	46.9 ± 2.0

^(1)^ Tests performed only within the group of data from this study, as the measurements of radioactivity of the same sample were conducted for two aging times, the alternative hypothesis is for being lesser. ^(2)^ In brackets is the sample size in a given group.

The result is that there is no statistically significant difference, since *p* value is always greater than α = 0.05. The construction of WMW test is that only when *p* < α, one is able to reject the null hypothesis in favor of the alternative one which in this case sounds “the average activity concentration of a given radionuclide in the group of cement samples sealed for 45 days is lesser than in the group of samples sealed for 30 days”. When one considers the whole groups of samples, with no division into cement types, the answer is obvious: one cannot reject the null hypothesis, and there is no visible difference, see also Figure 8.

Nonetheless, in the case of radionuclides from the uranium decay series, some shift reveals when considering activity concentration within groups CEM-I and CEM-IV. Under discussion would be whether the shift is statistically significant since one concludes from 14 vs. 5 cases (CEM-I) or 7 vs. 2 cases (CEM-IV), see also Table 7, where also some descriptive statistics has been presented, i.e., weighted mean and weighted deviation within each group under study.

Though the medians in all cases of activity concentrations are almost equal for ^40^K and ^232^Th, Figure 8, the medians within particular cement types may differ for ^226^Ra, which is the radionuclide of the interest when one spokes on secular equilibrium. Therefore, even though statistical tests for all cement types did not allow to claim that activity of ^226^Ra was less for 45-d aging than for 30-d aging, more data should be given for particular cement types, especially those with high concentrations of radium-226.

## 5. Conclusions

Based on the performed research, it was found that CM-8 and CM-15 pozzolanic cements containing silica dust, natural and industrial pozzolana and silica fly ash turned out to be the least safe in terms of radiological protection. It had the highest values of *Ra_eq_*, *D*, *E*, *H_ex_*, *H_in_*, *I_γ_*, and *I_α_* parameters; however, it was still beneath the accepted limits. The most safe materials in terms of radioactivity turned out to be Portland cements CM-1, CM-4, CM-5, CM-11, and CM-14, which did not contain any additives in the form of ashes or slags. Despite the fact that the use of mineral cement additives is justified from the point of view of reducing energy consumption, reducing CO_2_ emissions and increasing the durability of concrete, the negative impact of these treatments should also be taken into account.

As shown in the analyzes presented in this paper, waste additives in the form of fly ash and blast furnace slag may result in an increase in the concentration of natural radioactive radionuclides. The use of cements with this type of additives is therefore recommended in such buildings where the permanent contact of cement products with living organisms is limited. They can be successfully used in engineering facilities such as roads, bridges, viaducts, ports, etc. Based on the results of the analysis, it is recommended to use cements without additives in building elements where the contact of living organisms with cement products is permanent or long-lasting. Taking into account the potential composition variability of the waste substances themselves, their variable amount and variable residues after co-incineration, it is considered necessary to constantly monitor the concentration of natural radioactive radionuclides of potassium (^40^K), radium (^226^Ra) and thorium (^232^Th) in cements available on the market.

## Figures and Tables

**Table 1:** Types of cement commercially available on the Polish market with description and additives shown. The last column presents the number of samples, the aging time and the type of detector that collected the gamma spectra during the experiments.

Sample Name	Cement Type	Description	Additives	Number of Samples Taken, Detector (Maturing Time in Days)
CM-1	CEM-I	Portland cement	0–5% secondary ingredients	4, 3 × MAZAR (30 d), 1 × HPGe (45 d)
CM-2	CEM-II	Portland fly ash cement	21–35% silica fly ash, 0–5% secondary ingredients	4, 3 × MAZAR (30 d), 1 × HPGe (45 d)
CM-3	CEM-IV	Pozzolan cement	36–55% silica fume and natural and industrial pozzolan and silica fly ash, 0–5% secondary ingredients	4, 3 × MAZAR (30 d), 1 × HPGe (45 d)
CM-4	CEM-I	Portland cement	0–5% secondary ingredients	4, 3 × MAZAR (30 d), 1 × HPGe (45 d)
CM-5	CEM-I	Portland cement	0–5% secondary ingredients	4, 3 × MAZAR (30 d), 1 × HPGe (45 d)
CM-6	CEM-II	Portland fly ash cement	21–35% silica fly ash, 0–5% secondary ingredients	4, 3 × MAZAR (30 d), 1 × HPGe (45 d)
CM-7	CEM-II	Slag cement with fly ash	10–20% blast furnace slag, 10–20% silica fly ash, 0–5% secondary ingredients	4, 3 × MAZAR (30 d), 1 × HPGe (45 d)
CM-8	CEM-IV	Pozzolan cement	36–55% silica fume and natural and industrial pozzolan and silica fly ash, 0–5% secondary ingredients	4, 3 × MAZAR (30 d), 1 × HPGe (45 d)
CM-9	CEM-II	Portland composite cement	21–35% silica fly ash and limestone, calcium sulphate as binding time regulator	4, 3 × MAZAR (30 d), 1 × HPGe (45 d)
CM-10	CEM-II	Slag cement with fly ash	10–20% blast furnace slag, 10–20% silica fly ash, 0–5% secondary ingredients	4, 3 × MAZAR (30 d), 1 × HPGe (45 d)
CM-11	CEM-I	Portland cement	0–5% secondary ingredients	6, 4 × MAZAR (30 d), 2 × HPGe (45 d)
CM-12	CEM-II	Portland composite cement	21–35% silica fly ash and limestone, calcium sulphate as binding time regulator	6, 4 × MAZAR (30 d), 2 × HPGe (45 d)
CM-13	CEM-II	Portland fly ash cement	21–35% silica fly ash, 0–5% secondary ingredients	6, 4 × MAZAR (30 d), 2 × HPGe (45 d)
CM-14	CEM-I	Portland cement	0–5% secondary ingredients	1, 1 × MAZAR (30 d)
CM-15	CEM-IV	Pozzolan cement	36–55% silica fume and natural and industrial pozzolan and silica fly ash, 0–5% secondary ingredients	1, 1 × MAZAR (30 d)
TOTAL	15			60 (44 × MAZAR, 16×HPGe)

**Table 2:** Values of gamma radiation activity indices recommended by the European Commission [4].

Dose Criterion	0.3 mSv y^−1^	1.0 mSv y^−1^
Materials used in large amounts, e.g., cement,	*I_γ_* ≤ 0.5	*I_γ_* ≤ 1.0
Surface materials with limited usage, such as roof tiles, boards, panels	*I_γ_* ≤ 2.0	*I_γ_* ≤ 6.0

**Table 3:** Concentrations of radionuclides ^40^K, ^232^Th and ^40^K in samples of cement with designated uncertainties. Presented values are weighted means of 4–6 quantities obtained via measuring with MAZAR or HPGe devices (except for CM-14 and CM-15, see the text). Each individual uncertainty was estimated via the spectrometer software, while the values appearing beneath are weighted deviations.

Sample Name	Concentration Activity of Radionuclides (Bq kg^−1^)		
	^40^K	^226^Ra	^232^Th
CM-1	246 ± 9	24.8 ± 1.4	13.2 ± 0.8
CM-2	278 ± 32	62 ± 21	41 ± 12
CM-3	347 ± 40	75.6 ± 2.6	41.1 ± 1.9
CM-4	182 ± 14	33.9 ± 2.3	14.6 ± 1.1
CM-5	220 ± 33	25.1 ± 1.3	14.8 ± 1.1
CM-6	349 ± 33	49.1 ± 5.1	33.8 ± 5.6
CM-7	344 ± 8	64 ± 12	37.5 ± 5.8
CM-8	430 ± 17	73.6 ± 5.2	47.3 ± 3.2
CM-9	248 ± 9	34.8 ± 2.6	21.1 ± 0.8
CM-10	177 ± 3	47 ± 3	18.4 ± 0.5
CM-11	123 ± 14	21.7 ± 2.5	12.3 ± 2.3
CM-12	280 ± 27	53.1 ± 2.3	38.9 ± 4.2
CM-13	348 ± 28	54.0 ± 1.8	34.2 ± 2.8
CM-14	262 ± 39	27 ± 7	14 ± 3
CM-15	412 ± 45	75.7 ± 9.2	46.1 ± 6.3
Arithmetic mean	283 ± 89	48 ± 19	29 ± 13
Weighted mean	305 ± 78	56 ± 17	35 ± 11
Earth’s crust by average ^(1)^	400	35	30

^(1)^ According to UNSCEAR Report [1].

**Table 4:** Data received from other studies presenting measurements of radionuclides activity concentrations of ^226^Ra, ^232^Th, and ^40^K. Where it was possible, also information on the cement type was retrieved.

ISO Code	Cement Type (If Specified)	Concentration Activity of Radionuclides (Bq kg^−1^)			Literature Reference
		^40^K	^226^Ra	^232^Th	
ALB	CEM-I	169 ± 25	51.2 ± 5.5	16.1 ± 2.3	[37]
ALB	CEM-II	150 ± 20	51.0 ± 3.7	16.5 ± 3.6	
ALB	CEM-II	134 ± 12	46.2 ± 3.6	12.0 ± 3.1	
AUS	-	114	52	48	[16]
AUT	-	210	27	14	[17]
BRA ^(1)^	-	564	62	59	[20]
CHN	-	207.7	51.7	32	[21]
CMR	-	277	27	15	[66]
CUB	-	467	23	11	[38]
CYP	-	127	28	7	[42]
CZE(SVK)	-	157	12	18	[31]
DEU	-	325	15	23	[67]
EGY	-	73	19	15	[25]
EGY	-	82	35.6	43.2	[33]
ESP	-	182	34	13	[44]
EUR ^(2)^	-	216	45	31	[34]
FIN	-	251	40	20	[27]
GRC	-	257	85	19	[26]
GRC	CEM-I	154 ± 13	17 ± 1	15 ± 1	[36]
GRC	CEM-I	132 ± 13	15 ± 1	13 ± 2	
GRC	CEM-II	212 ± 15	91 ± 1	18 ± 3	
GRC	CEM-II	196 ± 17	89 ± 1	19 ± 3	
GRC	CEM-IV	244 ± 30	111 ± 17	19 ± 3	
IND	-	430	98	81	[18]
IND	-	177	24	20	[56]
IRN	-	291	40	29	[2]
ITA	-	316	46	42	[26]
ITA	-	357	41	63	[50]
ITA	-	218	38	22	[56]
JPN	-	139	36	21	[29]
KWT	-	240	13	9	[28]
LAO	-	116	38	14	[47]
MAR	-	238	31	19	[43]
MKD	-	264	42	28	[48]
MYS	-	204	81	59	[39]
NGA	-	114	8	2	[41]
NLD	-	230	27	19	[40]
NOR	-	259	30	19	[49]
PAK ^(2)^	-	273 ± 68	26.1 ± 5.6	28.7 ± 4.3	[29]
SEN	CEM-I	59.3 ± 7.3	136.0 ± 8.2	15.1 ± 0.9	[35]
SEN	CEM-II	81 ± 19	110 ± 29	12.0 ± 1.2	
SEN	CEM-III	80 ± 20	92 ± 17	12.2 ± 1.8	
SEN	CEM-IV	119 ± 12	8.1 ± 0.9	4.68 ± 0.78	
SVK	CEM-I	52.0	58.0	17.0	[32]
SVK	CEM-I	169.3	13.1	19.8	
SVK	CEM-II	314.6	10.8	32.8	
SVK	CEM-II	460	12.4	34.2	
SVK	CEM-III	417	16.7	37.5	
SVK ^(1)^	CEM-V	733	14.6	38.2	
SVK	CEM-I	228.3	9.3	18.2	
SVK	CEM-II	178.9	8.2	18.7	
SVK	CEM-II	146	12.1	16.0	
SVK	CEM-II	150.2	14.0	20.1	
SVK	CEM-III	111.3	21.6	22.9	
TUN	-	176	22	10	[29]
TUR	-	247	41	26	[30]
TUR	CEM-I	208 ± 16	34 ± 7	13 ± 2	[48]
TUR	CEM-II	221 ± 19	51 ± 12	18 ± 4	
TUR	CEM-IV	352 ± 49	45 ± 13	26 ± 5	
TUR ^(1)^	CEM-V	447	319	136	
TZA	-	228	46	28	[18]
YEM	-	428	40	25	[48]
mean ± SEM		241 ± 19	44 ± 6	25.3 ± 2.6	
(w.mean ± w.dev.)		(195 ± 88)	(83 ± 34)	(17.0 ± 4.7)	
Earth’s crust by average		400	35	30	

^(1)^ Extreme values were excluded from further statistical analysis. ^(2)^ The average of values presented in [34].

**Table 5:** Descriptive statistics and results of statistical tests for all the gathered data, both experimental data from this study, and from other studies.

Statistics	Concentration of ^40^K (Bq/kg)		Concentration of ^226^Ra (Bq/kg)		Concentration of ^232^Th (Bq/kg)	
	All Data	This Study	All Data	This Study	All Data	This Study
Mean ^(1)^	250	283	43	48	26	29
95% confidence interval of mean	(230, 270)	(252, 298)	(38, 47)	(43, 53)	(23, 28)	(26, 33)
Median	240	261	40	48	20	31
Std deviation	110	89	25	19	14	13
Weighted Deviation ^(1)^	93	78	27	17	13	11
Shapiro–Wilk	*p* = 0.063 ≈ α	*p* = 0.318 > α	*p* = 1.5 × 10^−5^ < α	*p* = 0.01 < α	*p* = 1.2 × 10^−6^ < α	*p* = 8.8 × 10^−4^ < α
Skewness coeff.	0.43	0.04	1.02	0.30	1.06	0.17
Correlation coeff. 90% confidence interval for ρ ^(2)^	K-Ra: ρ = 0.300 (0.09, 0.48)	K-Ra: ρ = 0.575 (0.41, 0.70)	Ra-Th: ρ = 0.436 (0.24, 0.59)	Ra-Th: ρ = 0.710 (0.58, 0.80)	K-Th: ρ = 0.524 (0.35, 0.66)	K-Th: ρ = 0.634 (0.49, 0.75)
Kendall rank	*p* = 1 × 10^−6^ < α	*p* = 1 × 10^−10^ < α	*p* = 2 × 10^−12^ < α	*p* = 1 × 10^−15^ < α	*p* = 2 × 10^−16^ < α	*p* = 1 × 10^−12^ < α
SUMMARY	Right-asymmetry, questionable normality	symmetry, normality	Extreme right-asymmetry, non-normality	Right-asymmetry, non-normality	Extreme right-asymmetry, non-normality	Right-asymmetry, non-normality

^(1)^ See also Table 3. ^(2)^ In the case of non-parametric tests, one has to estimate the interval via numeric methods, based on the sample size.

**Table 6:** Values of radiological parameters of the tested cements: radium equivalent activity index (*Ra_eq_*), gamma radiation dose level set out inside a premise (*D*), annual effective dose (*E*), external hazard index (*H_ex_*), internal hazard index (*H_in_*), gamma radiation activity index (*I_ă_*), the alpha radiation index (*I_á_*), all including the calculated uncertainties.

Sample Name	*Ra_eq_* (Bq kg^−1^)	*D* (nGy h^−1^)	*E* (mSv)	*H_ex_* (-)	*H_in_* (-)	*I_γ_* (-)	*I_α_* (-)
CM-1	62.4 ± 2.6	56.8 ± 2.3	0.276 ± 0.014	0.169 ± 0.072	0.235 ± 0.013	0.230 ± 0.011	0.124 ± 0.007
CM-2	142 ± 40	124 ± 34	0.61 ± 0.19	0.38 ± 0.13	0.55 ± 0.20	0.50± 0.17	0.31 ± 0.11
CM-3	160 ± 3	142.0 ± 3.4	0.70 ± 0.02	0.433 ± 0.010	0.637 ± 0.021	0.571 ± 0.015	0.378 ± 0.013
CM-4	68.7 ± 3.0	61.7 ± 2.8	0.302 ± 0.016	0.185 ± 0.009	0.277 ± 0.016	0.246 ± 0.013	0.170 ± 0.012
CM-5	63.2 ± 1.7	57.0 ± 1.3	0.279 ± 0.008	0.171 ± 0.006	0.238 ± 0.010	0.231 ± 0.006	0.125 ± 0.007
CM-6	124 ± 9	110.0 ± 7.4	0.539 ± 0.042	0.235 ± 0.028	0.467 ± 0.032	0.448 ± 0.038	0.245 ± 0.026
CM-7	143 ± 20	127 ± 18	0.62 ± 0.10	0.387 ± 0.063	0.56 ± 0.11	0.513 ± 0.080	0.318 ± 0.061
CM-8	174 ± 7	153.8 ± 5.7	0.754 ± 0.033	0.470 ± 0.022	0.669 ± 0.035	0.624 ± 0.024	0.368 ± 0.026
CM-9	84.0 ± 3.4	75.0 ± 3.1	0.368 ± 0.018	0.227 ± 0.011	0.32 ± 0.02	0.304 ± 0.014	0.174 ± 0.013
CM-10	87.1 ± 3.6	77.7 ± 3.2	0.381 ± 0.018	0.235 ± 0.011	0.36 ± 0.02	0.308 ± 0.014	0.236 ± 0.015
CM-11	48.4 ± 1.8	43.1 ± 1.5	0.21 ± 0.01	0.131 ± 0.007	0.189 ± 0.009	0.174 ± 0.009	0.108 ± 0.013
CM-12	130 ± 7	114 ± 6	0.56 ± 0.04	0.351 ± 0.026	0.495 ± 0.024	0.464 ± 0.036	0.266 ± 0.012
CM-13	129.7 ± 5.2	115.1 ± 4.6	0.564 ± 0.028	0.350 ± 0.018	0.496 ± 0.020	0.467 ± 0.026	0.270 ± 0.009
CM-14	67 ± 15	61 ± 13	0.30 ± 0.06	0.18 ± 0.04	0.254 ± 0.058	0.247 ± 0.052	0.135 ± 0.035
CM-15	173 ± 22	153 ± 19	0.751 ± 0.093	0.47 ± 0.06	0.673 ± 0.084	0.620 ± 0.078	0.379 ± 0.046
WEIGHTED AVERAGE	127 ± 36	115 ± 31	0.57 ± 0.17	0.32 ± 0.12	0.51 ± 0.16	0.47 ± 0.14	0.28 ± 0.09

## References

[1] United Nations Scientific Committee of Atomic Radiation (2000). Sources and Effects of Ionizing Radiation.

[2] Mehdizadeh S., Faghihi R., Sina S. (2011). Natural radioactivity in building materials in Iran. Nukleonika.

[3] International Commission on Radiological Protection, Pergamon Press (1994). Protection against 222Rn at Home and at Work.

[4] European Commission (1999). Radiological Protection Principles Concerning the Natural Radioactivity of Building Materials, Radiation Protection Report—RP-112.

[5] United Nations Scientific Committee on Effects of Atomic Radiation, United Nations Publications (2008). Effects of Ionizing Radiation: Report to the General Assembly with Scientific Annexes.

[6] (2014). Council Directive 2013/59/Euratom of 5 Dec. 2013 (2014) Laying Down Basic Safety Standards for Protection against the Dangers Arising from Exposure to Ionising Radiation, and Repealing Directives 89/618/Euratom, 90/641/Euratom, 96/29/Euratom, 97/43/ Euratom and 2003/122/Euratom. Off. J. Eur. Union.

[7] Przylibski T.A., Zebrowski A., Karpinska M., Kapa J., Kozak K., Mazur M., Grządziel D., Mamont-Cieśla K., Stawarz O., Kozłowska D. (2011). Mean annual ^222^Rn concentration in homes located in different geological regions of Poland—First approach to whole country area. J. Environ. Radioact..

[8] Tokonami S. (2020). Characteristics of Thoron (^220^Rn) and Its Progeny in the Indoor Environment. Int. J. Environ. Res. Public Health.

[9] Klepeis N.E., Nelson W.C., Ott W.R., Robinson J.P., Tsang A.M., Switzer P., Behar J.V., Hern S.C., Engelmann W.H. (2001). The National Human Activity Pattern Survey (NHAPS): A resource for assessing exposure to environmental pollutants. J. Expo. Sci. Environ. Epidemiol..

[10] Drzymała T., Zegardło B., Tofiło P. (2020). Properties of Concrete Containing Recycled Glass Aggregates Produced of Exploded Lighting Materials. Materials.

[11] Drzymała T., Jackiewicz-Rek W., Gałaj J., Šukys R. (2018). Assessment of mechanical properties of high strength concrete (HSC) after exposure to high temperature. J. Civ. Eng. Manag..

[12] Rudnik E., Drzymała T. (2018). Thermal behavior of polypropylene fiber-reinforced concrete at elevated temperatures. J. Therm. Anal. Calorim..

[13] Drzymała T., Jackiewicz-Rek W., Tomaszewski M., Kuś A., Gałaj J., Šukys R. (2017). Effects of High Temperature on the Properties of High Performance Concrete (HPC). Proc. Eng..

[14] Jackiewicz-Rek W., Drzymała. T., Kuś A., Tomaszewski M. (2016). Durability of high-performance concrete (HPC) subject to fire temperature impact. Arch. Civ. Eng..

[15] Kurdowski W. (2010). Chemia Cementu i Betonu [Cement and Concrete Chemistry].

[16] Beretka J., Mathew P. (1985). Natural Radioactivity of Australian Building Materials, Industrial Wastes and By-Products. Health Phys..

[17] Sorantin H., Steger F. (1984). Natural Radioactivity of building materials in Austria. Radiat. Prot. Dosim..

[18] Amasi A.I., Mtei K.M., Ijumba J.N., Jodłowski P., Chau N.D. (2014). Natural Radioactivity in Tanzania Cements and their Raw Materials. Res. J. Environ. Earth Sci..

[19] Azmary K.M., Ferdous J., Haque M.M. (2018). Natural Radioactivity Measurement and Assessment of Radiological Hazards in Some Building Materials Used in Bangladesh. J. Environ. Prot..

[20] Malanca A., Pessina V., Dallara G. (1993). Assessment of the natural radioactivity in the Brazilian state of Rio Grande do Norte. Health Phys..

[21] Lu X., Yang G., Ren C. (2012). Natural radioactivity and radiological hazards of building materials in Xianyang, China. Radiat. Phys. Chem..

[22] Zapotoczna-Sytek G., Mamont-Cieśla K., Tomasz Rybarczyk T. (2012). Naturalna promieniotwórczość wyrobów budowlanych, w tym autoklawizowanego betonu komórkowego (ABK) [Natural radioactivity of construction products, including autoclaved aerated concrete (AAC)]. Prz. Bud..

[23] Owczarek M. (2016). Promieniotwórczość materiałów budowlanych stosowanych w budownictwie (promieniowanie jonizujące) [Radioactivity of building materials used in construction (ionizing radiation)]. Inz. Bezpieczeństwa Obiektów Antropog..

[24] United Nations Scientific Committee on the Effects of Atomic Radiation (1982). Ionizing Radiation: Sources and Biological Effects.

[25] Fawizia A. (2007). Natural radioactivity levels in building materials used in Egypt. Radiat. Eff. Defects Solids.

[26] Trevisi R., Risica S., D’Alessandro M., Nuccetelli C. (2012). Natural radioactivity in building materials in the European Union: A database and an estimate of radiological significance. J. Environ. Radioact..

[27] Mustonen R. (1984). Natural radioactivity in and radon exhalation from Finnish building materials. Health Phys..

[28] Bou-Rabee F., Bem H. (1996). Natural radioactivity in building materials utilized in the State of Kuwait. J. Radioanal. Nucl. Chem..

[29] Khan K., Khan H.M. (2001). Natural gamma-emitting radionuclides in Pakistani Portland cement. Appl. Radiat. Isot..

[30] Turhan Ş., Baykan U.N., Sen K. (2008). Measurement of the natural radioactivity in building materials used in Ankara and assessment of external doses. J. Radiol. Protect..

[31] Eštoková A., Palaščáková L. (2013). Assessment of natural radioactivity levels of cements and cement composites in the Slovak Republic. Int. J. Environ. Res. Public Health.

[32] Eštoková A., Palaščáková P. (2013). Study of natural radioactivity of Slovak cements. Chem. Eng. Trans..

[33] El-Taher A., Makhluf S., Nossair A., Abdel Halim A.S. (2010). Assessment of natural radioactivity levels and radiation hazards due to cement industry. Appl. Radiat. Isot..

[34] Trevisi R., Leonardi F., Risica S., Nuccetelli C. (2018). Updated database on natural radioactivity in building materials in Europe. J. Environ. Radioact..

[35] Ndour O., Thiandoume C., Traore A., Cagnat X., Diouf P.M., Ndeye M., Ndao A.S., Tidjani A. (2020). Assessment of natural radioactivity and its radiological hazards in several types of cement used in Senegal. SN Appl. Sci..

[36] Papaefthymiou H., Gouseti O. (2008). Natural radioactivity and associated radiation hazards in building materialsused in Peloponnese, Greece. Radiat. Meas..

[37] Shala F., Xhixha G., Kaceli Xhixha M., Hasani F., Xhixha E., Shyti M., Sadiraj Kuqi M., Prifti D., Qafleshi M. (2017). Natural radioactivity in cements and raw materials used in Albanian cement industry. Environ. Earth Sci..

[38] Brígido Flores O., Montalván Estrada A., Rosa Suárez R., Tomás Zerquera J., Hernández Pérez A. (2008). Natural radionuclide content in building materials and gamma dose rate in dwellings in Cuba. J. Environ. Radioact..

[39] Chong C.S., Ahmad G.U. (1982). Gamma activity of some building materials in West Malaysia. Health Phys..

[40] Ackers J.G., Den Boer J.F., De Jong P., Wolschrijn R.A. (1985). Radioactivity and radon exhalation rates of building materials in The Netherlands. Sci. Total Environ..

[41] Ademola A.K., Abodunrin O.P., Afolake F.M., Omoboyede J.O. (2017). Assessment of natural radioactivity levels in cement samples commonly used for construction in Lagos and Ota environments, Nigeria. Elixir Nucl. Radiat. Phys..

[42] Michael F., Parpottas Y., Tsertos H. (2010). Gamma radiation measurements and dose rates in commonly used building materials in Cyprus. Radiat. Prot. Dosim..

[43] Kassi B., Boukhair A., Azkour K., Fahad M., Benjelloun M., Nourreddine A.-M. (2018). Assessment of Exposure Due to Technologically Enhanced Natural Radioactivity in Various Samples of Moroccan Building Materials. World J. Nucl. Sci. Technol..

[44] Ángel Sanjuán M., Suarez-Navarro J.A., Argiz C., Mora P. (2019). Assessment of radiation hazards of white and grey Portland cements. J. Radioanal. Nucl. Chem..

[45] Sharma A., Mahurc A.K., Yadavd M., Sonkawadee R.G., Sharmab A.C., Ramolad R.C., Prasadc R. (2015). Measurement of natural radioactivity, radon exhalation rate and radiation hazard assessment in Indian cement samples. Phys. Proc..

[46] Rizzo S., Brai M., Basile S., Bellia S., Hauser S. (2001). Gamma activity and geochemical features of building materials: Estimation of gamma dose rate and indoor radon levels in Sicily. Appl. Radiat. Isot..

[47] Xayheungsy S., Khiem L.H., Nam L.D. (2017). Radiation Dose Estimation of Cement Samples Used in Lao PDR. Commun. Phys..

[48] Özdis B.E., Cam N.F., Canbaz Öztürk B. (2017). Assessment of natural radioactivity in cements used as building materials in Turkey. J. Radioanal. Nucl. Chem..

[49] Stranden E., Berteiz L. (1980). Radon in dwellings and influencing factors. Health Phys..

[50] Sciocchetti G., Clemente G.F., Igrao G., Scacco F. (1983). Results of a survey on radioactivity of building materials in Italy. Health Phys..

[51] Poradnik ITB 455/2010 Badania Promieniotwórczości Naturalnej Wyrobów Budowlanych (Zastępujący Instrukcję ITB 234/2003) [ITB Guide 455/2010 Tests on Natural Radioactivity of Construction Products (Replacing ITB Instruction 234/2003)]. https://klient.itb.pl/itb/faces/pages/shop/shopping/paramProductView.xhtml?product=838.

[52] Drzymała T., Łukaszek-Chmielewska A., Lewicka S., Stec J., Piotrowska B., Isajenko K., Lipiński P. (2020). Assessment of the Natural Radioactivity of Polish and Foreign Granites Used for Road and Lapidary Constructions in Poland. Materials.

[53] Wallbrink P., Walling D.E., Quili H. (2007). Radionuclide Measurement Using HPGe Gamma Spectrometry. Handbook for the Assessment of Soil Erosion and Sedimentation Using Environmental Radionuclides.

[54] Organization of Economic Cooperation and Development (1979). Exposure to Radiation from Natural Radioactivity in Building Materials.

[55] Tufail M. (2012). Radium equivalent activity in the light of UNSCEAR report. Environ. Monit. Assess..

[56] Righi S., Bruzzi L. (2006). Natural radioactivity and radon exhalation in building materials used in Italian dwellings. J. Environ. Radioact..

[57] (2008). Uncertainty in Measurement. Part 3: Guide to the Expression of Uncertainty of Measurement.

[58] Cousineau D., Chartier S. (2010). Outliers detection and treatment: A review. Int. J. Psychol. Res..

[59] Veazie P.J. (2015). Understanding statistical testing. SAGE Open.

[60] Das K.R., Imon A.H.M.R. (2016). A Brief Review of Tests for Normality. Am. J. Theor. Appl. Stat..

[61] Dyckman T.R., Zeff S.A. (2019). Important Issues in Statistical Testing and Recommended Improvements in Accounting Research. Econometrics.

[62] Bewick V., Cheek L., Ball J. (2003). Statistics review 7: Correlation and regression. Crit. Care.

[63] University of Washington, Lectures of the Genome Sciences’ Faculty. https://www.gs.washington.edu/academics/courses/akey/56008/lecture/lecture7.pdf.

[64] Sawilowsky S.S. (1990). Nonparametric tests of interaction in experimental design. Rev. Edu. Res..

[65] R Documentation. http://www.sthda.com/english/.

[66] Ngachin M., Garavaglia M., Giovani C., Kwato Njock M.G., Nourreddine A. (2006). Assessment of natural radioactivity and associated radiation hazards in some Cameroonian building materials. Radiat. Meas..

[67] United Nations Scientific Committee on the Effect of Atomic Radiation (UNSCEAR) (1977). Sources and Effects of Ionizing Radiation.

[68] Ojovan M.I., Lee W.E., Ojovan M.I., Lee W.E. (2014). Naturally Occurring Radionuclides. An Introduction to Nuclear Waste Immobilisation.

[69] Schroeyers W. (2017). Naturally Occurring Radioactive Materials in Construction: Integrating Radiation Protection in Reuse (COST Action Tu1301 NORM4BUILDING).

[70] Kovacs T., Bator G., Schroeyers W., Labrincha J., Puertas F., Hegedus M., Doherty R. (2017). From raw materials to NORM by-products. Naturally Occurring Radioactive Materials in Construction.

[71] Nuccetelli C., Leonardi F., Trevisi R. (2015). A new accurate and flexible index to assess the contribution of building materials to indoor gamma exposure. J. Environ. Rad..

[72] Maringer F.J., Baumgartner A., Cardellini F., Cassette P., Crespo T., Dean J., Vodenik B. (2017). Advancements in NORM metrology—Results and impact of the European joint research project MetroNORM. Appl. Radiat. Isot..

[73] Barišić I., Netinger Grubeša I., Hackenberger Kutuzović B. (2017). Multidisciplinary approach to the environmental impact of steel slag reused in road construction. Road Mater. Pavement Des..

